# Coagulation during elective neurosurgery with hydroxyethyl starch fluid therapy: an observational study with thromboelastometry, fibrinogen and factor XIII

**DOI:** 10.1186/s13741-016-0046-z

**Published:** 2016-08-17

**Authors:** Caroline Ulfsdotter Nilsson, Karin Strandberg, Martin Engström, Peter Reinstrup

**Affiliations:** 1 Department of Anaesthesia and Intensive Care, Skåne University Hospital, Lund University, Lund, Sweden; 2 Department of Laboratory Medicine, Skåne University Hospital Malmö, Lund University, Malmö, Sweden; 3 Department of Anaesthesia and Intensive Care, Lund University, Lund, Sweden

**Keywords:** Factor XIII, Fibrinogen, Hydroxyethyl starch derivatives, Neurosurgery, Thromboelastography

## Abstract

**Background:**

Several studies have described hypercoagulability in neurosurgery with craniotomy for brain tumor resection. In this study, hydroxyethyl starch (HES) 130/0.42 was used for hemodynamic stabilization and initial blood loss replacement. HES can induce coagulopathy with thromboelastographic signs of decreased clot strength. The aim of this study was to prospectively describe perioperative changes in coagulation during elective craniotomy for brain tumor resection with the present fluid regimen.

**Methods:**

Forty patients were included. Perioperative whole-blood samples were collected for EXTEM and FIBTEM assays on rotational thromboelastometry (ROTEM) and plasma fibrinogen analysis immediately before surgery, after 1 L of HES infusion, at the end of surgery and in the morning after surgery. Factor (F)XIII activity, thrombin-antithrombin complex (TAT) and plasmin-α2-antiplasmin complex (PAP) were analysed in the 25 patients receiving ≥1 L of HES.

**Results:**

Most patients (37 of 40) received HES infusion (0.5–2 L) during surgery. Preoperative ROTEM clot formation/structure, plasma fibrinogen and FXIII levels were generally within normal range but approached a hypocoagulant state during and at end of surgery. ROTEM variables and fibrinogen levels, but not FXIII, returned to baseline levels in the morning after surgery. Low perioperative fibrinogen levels were common. TAT levels were increased during and after surgery. PAP levels mostly remained within the reference ranges, not indicating excessive fibrinolysis. There were no differences in ROTEM results and fibrinogen levels in patients receiving <1 L HES and ≥1 L HES.

**Conclusions:**

Only the increased TAT levels indicated an intra- and postoperative activation of coagulation. On the contrary, all other variables deteriorated towards hypocoagulation but were mainly normalized in the morning after surgery. Although this might be an effect of colloid-induced coagulopathy, we found no dose-dependent effect of HES. The unactivated fibrinolysis indicates that prophylactic use of tranexamic acid does not seem warranted under normal circumstances in elective neurosurgery. Individualized fluid therapy and coagulation factor substitution is of interest for future studies.

## Background

In neurosurgery, it is imperative to avoid intracranial bleeding. Perioperative bleeding can be associated with a number of factors including antihemostatic drugs and coagulation status but is also linked to the tumor’s vascularity, type, size and localization and the use of local hemostatics (Gerlach et al. 2004; Nittby et al. 2016). On the other hand, there is an increased risk of venous thromboembolism after elective neurosurgery (Collen et al. 2008). Hypercoagulation has been described in patients undergoing brain tumor surgery (Iberti et al. 1994; Nielsen et al. 2014).

In order to balance thrombosis and bleeding, we need to know the perioperative changes in coagulation. Among routine coagulation analyses are activated partial thromboplastin time (aPTT), prothrombin time (PT), platelet count and fibrinogen levels (Kozek-Langenecker 2010). Additional parameters that can be used are measurements of activated coagulation and fibrinolysis (e.g. thrombin-antithrombin complex (TAT) and plasmin-α2-antiplasmin complex (PAP)). Measurement of coagulation factor XIII (FXIII) levels or activity is becoming increasingly recognized as important during surgery (Levy and Greenberg 2013). Viscoelastic instruments such as thromboelastography (TEG) and rotational thromboelastometry (ROTEM) are point-of-care instruments that are helpful for quickly assessing global hemostatic function in whole blood and for guiding treatment of bleeding (Afshari et al. 2011).

Hydroxyethyl starch (HES) is a colloid solution that can be used to replace initial blood loss and to treat hypovolemia during elective surgery. However, HES can induce a hypocoagulable state by diluting fibrinogen and FXIII, as well as it affects fibrin polymerization and clot structure (Fenger-Eriksen et al. 2009). After the publication of several large randomized controlled trials indicating a risk of kidney injury in critically ill patients receiving HES, its use has been diminished over the last few years. However, there is still controversy as to whether HES should be avoided in all clinical situations, and evidence of HES-induced kidney injury in the perioperative setting is lacking (Greenberg and Tung 2015; European Medicines Agency 2014). The European Medicines Agency currently states that HES may be used to treat acute hypovolemia, but not in patients with sepsis, critical illness, severe coagulopathy and renal injury (European Medicines Agency 2014).

The local routine at our hospital was to use HES (130/0.42) for hemodynamic stabilization and initial blood loss replacement during elective brain tumor resection. The aim of this prospective observational study was to describe perioperative coagulation changes with ROTEM, TAT, PAP, FXIII activity and fibrinogen levels in these patients.

## Methods

### Ethical approval and patients

The study was approved by the regional ethics committee (Lund, Protocol DNR 2012/43) and was performed at Skåne University Hospital in Lund, Sweden. The study included 40 patients undergoing elective craniotomy and tumor resection.

All patients were >18 years old and gave written consent to participate. Patients with a known congenital hemophilic or thrombophilic coagulation disorder and/or who were treated with anticoagulants/antiplatelet agents within 5 days before surgery were not enrolled. Preoperative coagulation tests PT, aPTT and platelet count were not routinely analysed in patients with no history of bleeding disorders (according to previous findings (Seicean et al. 2012)). Patients with abnormal aPTT and/or PT and a platelet count below the reference range were excluded. Patients with abnormal serum-creatinine (>90 μmol/L for women and >105 μmol/L for men) were also excluded. For logistical reasons, only patients scheduled for surgery in the morning were chosen to participate. Patients meeting the inclusion criteria were enrolled consecutively from February through May 2012.

The majority of patients had dexamethasone treatment prior to surgery in order to reduce tumor edema. All patients received a preoperative prophylactic dose of peroral rifampicin. Standard anaesthesia with fentanyl, propofol, isoflourane and rocuronium was used.

All patients received mechanical calf compression thromboprophylaxis during surgery and 24 h postoperatively. The fluid protocol included isotonic saline infusion as maintenance fluid (1.5–2.0 mL/kg/h). Bleeding (200–300 mL) was initially substituted with saline (1:2 bleeding to saline). Additional bleeding was substituted with HES (Venofundin® 60 mg/mL [6% hydroxyethyl, molecular weight (MW) 130 kDa, substitution 0.42, in saline solution, Braun, Melsungen Germany], 1:1 bleeding to HES), with a maximum dose of 30 mL/kg. HES was also used to keep mean arterial blood pressure (MAP) at >65 mmHg. Red blood cell transfusion was given when hemoglobin levels declined below 95–100 g/L. Blood loss of more than 30 % of calculated blood volume was substituted with red blood cells, fresh frozen plasma and platelet concentrates.

Local hemostatics (SurgiSeal®, Adhezion Biomedica, PA, USA, and TachoSil®, Takeda, High Wycomb, UK) were applied at the discretion of the surgeon. The coagulation assays TAT, PAP, fibrinogen and FXIII were performed in a batch after the completion of the study enrolment. TAT, PAP and FXIII were analysed in a subset of patients (those receiving ≥1 L HES) due to the initial plan to focus in depth coagulation studies on these patients (more homogenous with respect to HES volumes administered). Perioperative ROTEM analyses, especially abnormal EXTEM-MCF and FIBTEM-MCF, were shown to the anaesthetist in charge, who evaluated the hemostatic status together with the surgeon to decide whether plasma, platelet transfusion or fibrinogen concentrate were to be administered. Apart from this safety measure of informing the anaesthetist in charge, there was no intervention in the management of patients.

### Blood sampling

Arterial blood samples were drawn from an indwelling radial arterial catheter with continuous flushing and a sampling membrane which eliminates the need for disposing blood samples.

Blood sampling for the study was performed before surgery (after the induction of anaesthesia, baseline), after 1 L of HES infusion (only analysed in patients receiving ≥1 L HES), at the end of surgery and in the morning after surgery (the first postoperative day).

Blood was collected in citrated tubes (BD Vacutainer® 4.5 mL 0.129 M for laboratory plasma analysis and 2.7 mL 0.109 M for ROTEM analysis). The blood samples intended for laboratory plasma analysis were immediately centrifuged for 20 min at 2000 rpm at a temperature of 20 °C to obtain the plasma fractions. Plasma vials for the separate tests (TAT, PAP, fibrinogen and FXIII) were frozen and stored in a −85 °C freezer until analysis.

### Surgical blood loss

The amount of bleeding during surgery was assessed by weighing sponges and measuring losses in the suction device.

### ROTEM

ROTEM analysis (TEM International GmbH, Munich, Germany) was performed according to the manufacturer’s instructions with EXTEM (tissue factor activation) and FIBTEM (tissue factor activation and platelet inhibition) reagents. The parameters obtained with EXTEM were clotting time (CT), clot formation time (CFT), *α*-angle and maximum clot firmness (MCF), whereas MCF was obtained with FIBTEM. Reference intervals provided by the ROTEM manufacturer were used: EXTEM: CT 38–79 s, CFT 34–159 s, *α*-angle 63–83°, MCF 50–72 mm, and FIBTEM: MCF 9–25 mm. A ROTEM variable within the reference interval indicated normal coagulability, whereas a variable outside the reference interval indicated increased or decreased coagulability.

### Laboratory plasma analyses

Fibrinogen was measured with a photometric assay (Multifibren U, Siemens, AG, Gerlangen, Germany). Thrombin (50 U/mL) was added in excess to plasma samples. Clotting time was recorded with an automated coagulometer (Symex CA 7000, Siemens AG, Gerlangen, Germany) and compared to clotting times with known fibrinogen concentrations. The reference interval for fibrinogen is 2–4 g/L, according to the manufacturer.

FXIII activity was determined with the automated Berichrom FXIII (Siemens Healthcare Diagnostics, Marburg, Germany) method, on the BCS-XP Coagulation analyser (Siemens Healthcare Diagnostics, Marburg, Germany). FXIII in the plasma sample is converted to FXIIIa after the addition of thrombin. FXIIIa is detected in an enzymatic reaction in which ammonia is released. The absorbance at 340 mm is proportional to the FXIIIa activity in the sample. The reference interval in healthy adults is 0.70–1.40 kIU/L according to the manufacturer.

TAT was measured using Enzygnost TAT micro (Siemens Healthcare Diagnostics, Marburg, Germany), a solid-phase enzyme-linked immunoassay (ELISA). The reference interval in healthy adults is 1.0–4.1 μg/L (2.5–97.5 percentile, *n* = 196) according to the manufacturer.

PAP was determined using DRG PAP micro ELISA (DRG Instruments GmbH, Marburg, Germany), a solid-phase ELISA based on a sandwich principle. The reference interval in healthy adults is 120–700 μg/L (2.5–97.5 percentile, *n* = 466) according to the manufacturer.

### Statistical analysis

Data was processed using Microsoft Excel® and GraphPad Prism. Results are presented as median and range. The Wilcoxon matched-pairs signed rank test was performed to find changes in the variables from baseline compared to after 1 L HES, at the end of surgery and in the morning after surgery.

Statistics were also performed with patients divided into groups receiving a low dose (<1 L) or higher dose (≥1 L) of HES in order to investigate a possible dose-response. The Mann-Whitney *U* test for unpaired data was used to detect differences between the groups at baseline, at the end of surgery and in the morning after surgery.

After Bonferroni correction for the number of significance tests per each variable (*n* = 6), a *P* value of <0.0083 (0.05/6) was considered statistically significant at a *P* < 0.05 level.

For five measured FXIII activity levels, the activity was >1.299 kIU/L. This right-truncated data (>1.299) was treated as =1.299 in statistical calculations and in graphs.

Fibrinogen and FXIII levels were correlated with FIBTEM-MCF levels using the Spearman rank correlation.

## Results

### Study population and clinical data

The study included 40 patients (16 males and 24 females), aged 35–81 years (median 56 years), with median BMI 25 (range 17.5–39). Meningioma was the most common diagnosis (18 patients); other tumor types included metastasis, astrocytoma, schwannoma, glioblastoma, ependymoma, craniopharyngioma and chordoma. Operation times ranged from 2 to 10 h, with a median time of 5 h.

Preoperative hemoglobin levels were 128 g/L (range 96–169 g/L). Median bleeding during surgery was 450 mL, ranging from 50 to 2500 mL. Nine patients had bleeding of ≥1 L during surgery. Of the six patients with bleeding of >1 L, all but one had surgery for meningioma. Fifteen patients received <1 L HES (between 500 and 800 mL), including three patients who did not receive any HES at all. Twenty-five patients received ≥1 L HES, with only one patient receiving a large volume of 2 L. Twelve patients were transfused with blood components during surgery (red blood cells, plasma and/or platelets). One patient was given one dose of tranexamic acid during surgery. Seven patients were also given 5 % albumin in waiting for plasma. No patient needed reoperation because of postoperative hematoma. Characteristics of the patients divided into groups (<1 L HES and ≥1 L HES) are seen in Table 1.

**Table 1:** Group characteristics

	<1 L HES	≥1 L HES
Number of patients	15	25
Age (years)	57 (31–79)	55 (35–81)
Operation duration (h)	3.5 (2–7)	6 (2–10)
Bleeding during surgery (mL)	200 (50–2000)	700 (70–2500)
Number of patients receiving blood component therapy (red blood cells, plasma and/or platelets) during surgery	2	10
Reoperation due to hematoma	0	0

Group characteristics for patients receiving <1 L HES and ≥1 L HES. Median (range)

### ROTEM

Preoperative hypercoagulation as seen with ROTEM variables was only found in one patient with a shortened CT (36 s). Signs of preoperative decreased coagulability were seen in eight patients with ROTEM. Five patients had low alpha angle and/or low FIBTEM-MCF. Three patients had impaired CFT, alpha angle, MCF and FIBTEM-MCF, and two of these had prolonged CT.

**Fig. 1:**
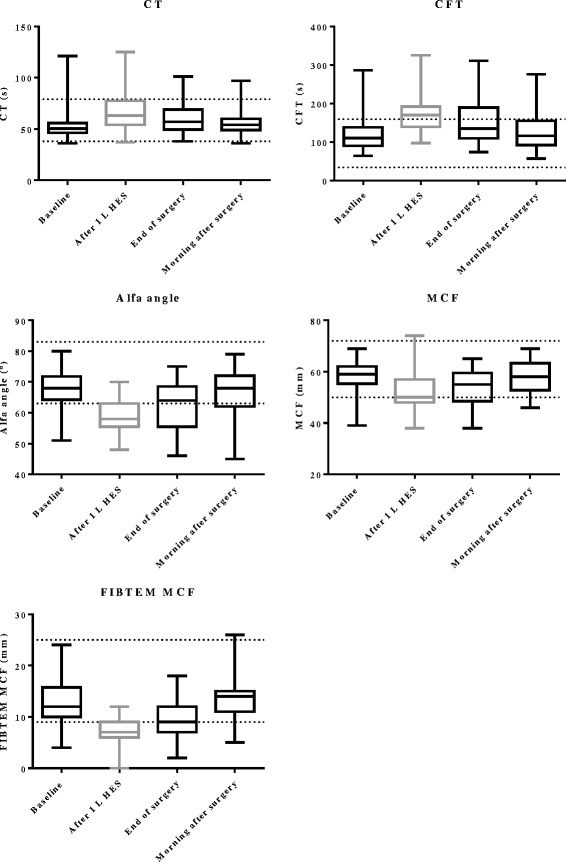
Perioperative ROTEM variables. *Black boxes* are all patients, whereas the *grey box* in each graph represents only the patients receiving ≥1 L HES. The reference ranges for the variables are indicated by *horizontal lines* from the *Y*-axis

Statistical analysis for all patients (*n* = 40) showed that all ROTEM variables (CFT, alpha angle, MCF and FIBTEM-MCF) except for CT were changed towards impaired coagulation at the end of surgery compared to baseline (*P* < 0.0001, Table 2, Fig. 1). All ROTEM variables were also impaired after administration of 1 L HES compared to baseline (*P* < 0.0001). ROTEM variables returned to baseline values in the morning of the first postoperative day.

**Table 2:** Descriptive data and statistics

	Reference interval	Baseline	After 1 L HES (patients receiving ≥1 L HES)	*P* value	End of surgery	*P* value	Morning after surgery	*P* value
				Baseline vs after 1 L HES (patients receiving ≥1 L HES)		Baseline vs end of surgery		Baseline vs morning after surgery
Analysed in all patients (*n* = 40)								
CT (s)	38–79	50.5 (36–121)	63 (37–125)	*<0.0001*	57 (38–101) [3]	0.027	54 (36–97) [5]	0.41
CFT (s)	34–159	110.5 (64–286)	170 (97–325)	*<0.0001*	135 (74–311) [3]	*<0.0001*	116 (57–276) [5]	0.13
Alpha angle (°)	63–83	68 (51–80)	58 (48–70)	*<0.0001*	64 (46–75) [3]	*<0.0001*	68 (45–79) [5]	0.034
MCF (mm)	50–72	59 (39–69)	50 (38–74)	*<0.0001*	55 (38–65) [3]	*<0.0001*	58 (46–69) [6]	0.24
FIBTEM-MCF (mm)	9–25	12 (4–24)	7 (0–12)	*<0.0001*	9 (2–18) [5]	*<0.0001*	14 (5–26) [5]	0.051
Fibrinogen (g/L)	2–4	1.9 (0.9–3.3)	1.4 (0.6–2.8)	*<0.0001*	1.5 (0.6–2.8)	*<0.0001*	2.4 (0.6–3.4) [5]	0.012
Analysed in patients receiving ≥1000 mL HES (*n* = 25)								
FXIII (kIU/L)	0.7–1.4	0.9 (0.43–>1.30)	0.62 (0.32–1.16)	*<0.0001*	0.68 (0.39–1.19) [3]	*<0.0001*	0.84 (0.44–>1.30) [3]	*<0.0001*
TAT (μg/L)	1.0–4.1	4.4 (2–68) [1]	7.2 (2–98)	0.0087	23.4 (2–142) [3]	*0.0004*	8.1 (3–449) [2]	*0.0032*
PAP (μg/L)	120–700	541.3 (275–1997)	419.5 (218–1478)	*<0.0001*	392.4 (276–1339) [3]	*<0.0001*	481.0 (288–2297) [2]	0.80

Data is presented as median values (range) [missing]. Italicized *P* values are statistically significant (*P* < 0.0083)

*HES* hydroxyethyl starch, *CT* clotting time, *CFT* clot formation time, *MCF* maximum clot firmness, *TAT* thrombin-antithrombin complex, *PAP* plasmin-α2-antiplasmin complex, *FXIII* factor XIII

There were no statistically significant differences between the groups (patients receiving <1 L HES and ≥1 L HES) at baseline, at the end of surgery and in the morning after surgery for any of the ROTEM variables (*P* > 0.0083).

### Laboratory plasma analyses

**Fig. 2:**
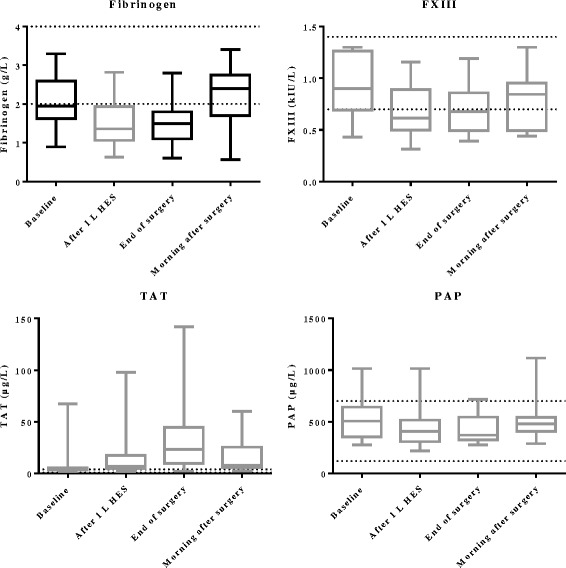
Perioperative fibrinogen, FXIII, TAT and PAP levels. *Black boxes* are all patients, whereas the *grey boxes* are the patients receiving ≥1 L HES. The reference ranges for the variables are indicated by *horizontal lines* from the *Y*-axis. In the FXIII graph, values >1.299 were plotted as 1.299. In the TAT graph, one value was omitted from the morning after surgery (449 μg/L). In the PAP graph, one patient was omitted (PAP levels 1997.4–1477.9-1339.3–2296.7 μg/L)

Twenty patients had low fibrinogen (<2.0 g/L) before surgery. At the end of surgery, 21 of 40 patients had a fibrinogen level of ≤1.5 g/L. Fibrinogen decreased from the baseline median of 1.9 to 1.5 g/L by the end of surgery (*P* < 0.0001, Table 2, Fig. 2) but increased again until the first morning after surgery (median 2.4 g/L). Fibrinogen was decreased compared to baseline after the administration of 1 L HES (*P* < 0.0001). All patients with low preoperative FIBTEM-MCF (<9 mm, *n* = 5) had low fibrinogen levels (0.9–1.4 g/L). There were no differences in fibrinogen levels between the groups (patients receiving <1 L HES or ≥1 L HES) at baseline, at the end of surgery and in the morning after surgery (*P* > 0.0083).

In the 25 patients who received ≥1 L HES (*n* = 25), six patients had low preoperative FXIII which remained below the reference range during the observation period. FXIII activity was decreased compared to baseline after 1 L HES and at the end of surgery, and it remained decreased in the morning after surgery (*P* < 0.0001, Table 2, Fig. 2).

TAT levels (>4.1 μg/L) were significantly elevated at the end of surgery and remained elevated the morning after surgery (Table 2, Fig. 2). TAT levels were borderline significantly elevated after 1 L HES (Table 2). PAP was decreased after 1 L HES and at the end of surgery but returned to preoperative levels in the morning after surgery (Table 2, Fig. 2). However, PAP mainly remained within the normal reference range.

### Variable correlation

FXIII activity correlated poorly with FIBTEM-MCF with a correlation coefficient of 0.54 (*P* < 0.01). Fibrinogen correlated better with FIBTEM-MCF with a correlation coefficient of 0.70 (*P* < 0.01).

## Discussion

Increased (“hyper”) or decreased (“hypo”) coagulability was defined as variables outside the reference intervals (Görlinger et al. 2013). Based on this definition, the preoperative coagulation state in our neurosurgical patients mostly appeared normal on ROTEM but approached a hypocoagulable state during surgery and at the end of surgery, only to return to baseline levels in the first postoperative morning (or, for CT, at end of surgery). Signs of perioperative hypercoagulability were uncommon.

Like our results, previous viscoelastic studies of elective neurosurgical patients describe both a mainly normal preoperative coagulation status in accordance with our findings (Goobie et al. 2001; El Kady et al. 2009; Lindroos et al. 2014) but, unlike our findings, an increased coagulability (varying definitions) during and after surgery (Nielsen et al. 2014; Goobie et al. 2001; El Kady et al. 2009; Abrahams et al. 2002; Goh et al. 1997). Two studies found that patients who developed a postoperative hematoma had impaired coagulation as compared to patients who did not develop a hematoma (El Kady et al. 2009; Goh et al. 1997). Thus, the impaired coagulation we identified during surgery might increase the risk for postoperative intracranial hematomas. Explanations for this impaired coagulation could be blood loss, coagulation factor consumption or dilution by fluids including HES-induced coagulopathy. A possible HES effect needs to be validated in a randomized trial comparing HES to another fluid regime including its implications for bleeding and thrombotic events in neurosurgery.

Although modern starches (such as 130/0.4) seem to have little effect on perioperative bleeding in major surgery (Kozek-Langenecker 2015), a dose-response of the negative impact on clot strength by HES 130/0.4 has previously been described, primarily by in vitro studies (Hartog et al. 2011). In the present study, we did not see a dose-response of HES on coagulation (ROTEM and fibrinogen levels), as we compared patients who received either <1 L HES or ≥1 L HES; however, this is a small study in which almost all patients received HES, making conclusions about a dose-response difficult. Although our study is underpowered to detect a correlation between the different variables and clinical bleeding/postoperative complications, no patient needed reoperation due to hematoma. Median bleeding volume was higher in patients who received the higher doses of HES, but probably reflects that more bleeding prompted more volume replacement. Of the six patients who bled >1 L, all but one had meningioma surgery. Meningiomas are known to be highly vascular and bleeding can be a problem during resection and postoperatively (Gerlach et al. 2004).

Other studies have looked at HES in neurosurgery. A study by Lindroos et al. that included 30 patients detected signs of impaired ROTEM FIBTEM clot formation and strength during neurosurgery with HES infusion (130/0.4), but not with Ringer’s acetate (Lindroos et al. 2014). ROTEM EXTEM was unaffected. The mean HES volume was 440 mL, which is less than the HES volumes that was used for our patients and could explain why we saw a more pronounced impaired coagulation. Two retrospective studies of more than 4000 patients (Feix et al. 2015) and more than 40,000 patients (Jian et al. 2014) did not find an association between the use of HES 130/0.4 (average volume 700 mL and median volume 500 mL, respectively) and a risk of reoperation for intracranial hematoma after craniotomy. As mentioned, evidence so far do not suggest that modern day HES increases bleeding in other types of major surgery. However, this fluid still impairs fibrin polymerization and clot strength (Kozek-Langenecker 2015) and this effect in neurosurgery has not been properly evaluated. Currently, the role of HES in the perioperative setting is still largely unknown, and further studies regarding the safety, timing and choice of colloids are necessary (Coriat et al. 2014). Furthermore, preoperative coagulation testing could possibly indicate which patients can tolerate larger volumes of HES and which patients should be given HES or other colloids with care, but this “dilutive capacity strategy” in patients needs to be tested in prospective studies.

Preoperative low fibrinogen (<2 g/L) was common in the present study and decreased further during and at the end of surgery but returned to baseline levels on the first postoperative morning. HES is known to influence photometric methods for measuring fibrinogen (Fenger-Eriksen et al. 2010) with falsely high values, so fibrinogen levels could have been even lower in our study. There is no specific recommendation for fibrinogen levels during intracranial surgery, but a perioperative low fibrinogen (<1.5 g/L) has previously been associated with an increased risk of postoperative intracranial hematoma (Gerlach et al. 2002). A more recent retrospective study suggests targeting a perioperative fibrinogen level >2 g/L to avoid postoperative hematomas (Wei et al. 2015). The importance of fibrinogen has also been shown by Adelmann et al., who studied 290 patients undergoing elective neurosurgery and found lower fibrinogen levels (mean 1.7 g/L) at the end of surgery in patients who developed postoperative hematoma compared to patients who had no hematoma (fibrinogen mean 2.4 g/L) (Adelmann et al. 2014). It seems that low fibrinogen in this type of surgery can be dangerous, and as we found low levels to be common (in our study, 21 of 40 patients had a fibrinogen level of ≤1.5 at the end of surgery), how and when to prophylactically treat a low fibrinogen level in neurosurgery remains to be studied.

FIBTEM-MCF can be an indicator of low fibrinogen; we found a correlation between FIBTEM-MCF and fibrinogen (*R* = 0.7), which is comparable to previous findings (Solomon et al. 2011). This test is however also affected by other proteins such as FXIII (Schöchl et al. 2011), even though we found the correlation between FXIII and FIBTEM-MCF to be poor.

Acquired FXIII deficiency and substitution of FXIII is increasingly studied during surgical procedures (Levy and Greenberg 2013; Gerlach et al. 2009), much due to better FXIII assays. A large observational study of more than 1200 neurosurgical intracranial procedures found an increased risk of postoperative hematoma in patients who had low postoperative FXIII activity (Gerlach et al. 2000). A subsequent prospective study of more than 800 patients has found an association between decreased perioperative FXIII (FXIII activity of <60 %, which corresponds to <0.6 kIU/L) and an increased risk of postoperative intracranial hematoma (Gerlach et al. 2002). Of the 25 patients receiving ≥1 L HES in our study, 11 patients had FXIII activity of <0.6 kIU/L after HES infusion and FXIII activity was still low on the first postoperative morning. However, in the study by Adelmann et al. (mentioned above), FXIII activity was not lower in elective neurosurgical patients who developed postoperative hematoma compared to patients who did not (Adelmann et al. 2014). Many questions still remain to be answered on how and when to treat surgical patients with FXIII concentrates. Although FXIII supplementation was not beneficial in cardiac surgery (Karkouti et al. 2013), this does not necessarily translate to neurosurgery.

TAT is a marker for the generation of thrombin and thus for coagulation activation (Amiral and Fareed 1996), but unlike ROTEM variables, it does not provide information on clot structure. Elevated TAT levels indicate procoagulant plasma reactions during surgery as seen in our study, probably as a response to the surgical trauma. This is supported by two previous studies who also found elevated TAT levels during neurosurgery (Fujii et al. 1994; Heesen et al. 1997).

PAP is a marker for plasmin generation and an indicator of fibrinolytic activation (Montes et al. 1996). Unlike in a previous neurosurgical study that found increased PAP during surgery (Fujii et al. 1994), the present study found perioperative levels within the reference range. Our results therefore do not advocate the prophylactic use of tranexamic acid for fibrinolysis inhibition, as has been suggested for many types of surgery (Ker et al. 2012).

The strengths of this study are the meticulous repeated blood sampling and the inclusion of both ROTEM and advanced plasma analysis of hemostasis. Limitations are primarily the small study population and the lack of a control group (no HES or another colloid).

## Conclusions

In conclusion, perioperative signs of increased coagulability were extremely uncommon in this prospective observational study. Only TAT levels indicated activation of coagulation. PAP levels showed no fibrinolytic activation, thus not advocating routine prophylactic use of tranexamic acid. There was an overall impaired coagulation during and at the end of surgery compared to the pre-surgery coagulation status, which was mainly normalized the day after surgery. The impaired coagulation could possibly be an effect of HES but needs to be further studied in randomized controlled studies. A more advanced perioperative coagulation testing method with thromboelastometry, fibrinogen levels and FXIII activity could help to reduce bleeding by an individualized regimen of fluids, transfusion and coagulation factor substitution, but this requires further studies.

## Abbreviations

APTT, activated thromboplastin time; CFT, clot formation time; CT, clotting time; FXIII, factor XIII; HES, hydroxyethyl starch; MCF, maximum clot firmness; PAP, plasmin-antiplasmin complex; PT, prothrombin time; ROTEM, rotational thromboelastometry; TAT, thrombin-antithrombin complex; TEG, thromboelastography
